# A novel universal real-time PCR system using the attached universal duplex probes for quantitative analysis of nucleic acids

**DOI:** 10.1186/1471-2199-9-54

**Published:** 2008-06-04

**Authors:** Litao Yang, Wanqi Liang, Lingxi Jiang, Wenquan Li, Wei Cao, Zoe A Wilson, Dabing Zhang

**Affiliations:** 1 GMO detection laboratory, SJTU-Bor Luh Food Safety Center, School of life Science and Biotechnology, Shanghai Jiao Tong University, 800 Dongchuan Road, Shanghai 200240, PR China; 2 Amergen Biomed Co., Ltd., 22B, 3131 Hong Mei Rd., Shanghai 201103, PR China; 3 Plant Sciences Division, School of Biosciences, University of Nottingham, Sutton Bonington Campus, Loughborough, Leicestershire LE12 5RD, UK

## Abstract

**Background:**

Real-time PCR techniques are being widely used for nucleic acids analysis, but one limitation of current frequently employed real-time PCR is the high cost of the labeled probe for each target molecule.

**Results:**

We describe a real-time PCR technique employing **a**ttached **u**niversal **d**uplex **p**robes (AUDP), which has the advantage of generating fluorescence by probe hydrolysis and strand displacement over current real-time PCR methods. AUDP involves one set of universal duplex probes in which the 5' end of the fluorescent probe (FP) and a complementary quenching probe (QP) lie in close proximity so that fluorescence can be quenched. The PCR primer pair with attached universal template (UT) and the FP are identical to the UT sequence. We have shown that the AUDP technique can be used for detecting multiple target DNA sequences in both simplex and duplex real-time PCR assays for gene expression analysis, genotype identification, and genetically modified organism (GMO) quantification with comparable sensitivity, reproducibility, and repeatability with other real-time PCR methods.

**Conclusion:**

The results from GMO quantification, gene expression analysis, genotype identification, and GMO quantification using AUDP real-time PCR assays indicate that the AUDP real-time PCR technique has been successfully applied in nucleic acids analysis, and the developed AUDP real-time PCR technique will offer an alternative way for nucleic acid analysis with high efficiency, reliability, and flexibility at low cost.

## Background

PCR is a powerful tool for the amplification of minimal amounts of initial target genetic sequences [1,2], and it has been widely used for quantitative analysis of nucleic acids in medical diagnostics, drug discovery, virus detection, environmental monitoring, gene expression analysis and the detection of genetically modification organisms (GMO) [3-5]. Fluorescent quantitative PCR (FQ-PCR) can quantify initial target molecules by detecting the amplicon accumulation with fluorescently-tagged probes at each reaction cycle. Because of its ease of use, high throughput ability, decreased post-PCR manipulation, and lack of cross-contamination of PCR amplicons, FQ-PCR method is becoming the new gold standard method for nucleic acid quantification. Currently, several fluorogenic signal reagents have been developed and applied in nucleic acid analyses, for instance, DNA-binding dyes [6,7], FRET probes [8], TaqMan probes [9,10], molecular beacons [11,12] and their derivatives (Amplifluor, Sunrise, Amplisensor, and scorpion primers) [13-16], double-stranded probes [17], adjacent probes [18], iFRET [19], universal template probe [20], and aQRT-PCR [21]. Probes based on the FRET technique, such as TaqMan, Amplisensor, molecular beacon, and UT probes, rely on the principle that a donor fluorophore molecule absorbs excitation energy and delivers this energy via dipole-dipole interaction to an acceptor fluorophore when the donor and acceptor molecules are sufficiently close to one another [22]. When the donor fluorophore and acceptor fluorophore are separated in the reaction by cleavage, the change in secondary structure or strand displacement of the fluorescent probe will result in increased fluorescence signal [23-25]. In these real-time PCR assays, either dual-labeled probes or primers have to be synthesized, which limits the wide application of these methods due to incomplete quenching, complicated synthesis and labeling, and high costs [26].

Herein we report a novel set of fluorescent signal device named **A**ttached **U**niversal **D**uplex **P**robes (AUDP) with a single-labeled fluorescent probe (FP) with universal template (UT) sequence and a quenching probe (QP). In the AUDP PCR assay, the fluorescent signals are likely to be generated by means of DNA strand displacement and hydrolysis of the duplex probes. AUDP can not only be used for different target DNA sequences in single PCR assays, but also in duplex PCR assays with higher fluorescent intensity. Moreover, amplified target DNA fragments as long as 1.5 kilo base pairs can be detected with high efficiency using this method. These enable AUDP based real-time PCR assay to be a flexible, reproducible, inexpensive, and effective method for nucleic acid quantification.

## Results

### The fluorescent signal generated principle of AUDP PCR assay

**Figure 1:**
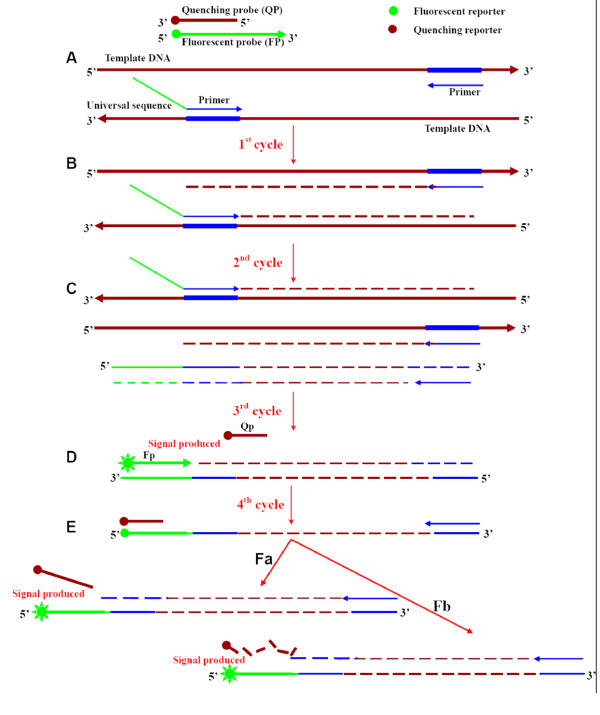
**Schematic representation of the mechanism of AUDP PCR assay**. (A) The structure of the duplex probes and modified target specific primers; (B) The first cycle of PCR amplification; (C) The second cycle of amplification. (D) The third cycle of amplification and fluorescent signal generation; (E), (Fa) and (Fb), The fourth cycle of amplification and fluorescent signal generation.

The basic goal of many fluorescence-based quantitative PCR procedures is to produce signal indicative of the presence of double-stranded DNA during PCR amplification. In this study, we have developed a new combined method for fluorescent signal generation, termed AUDP and illustrated in Figure 1. A complete AUDP PCR assay is composed of a target DNA-specific primer pair, with one primer containing an attached UT sequence, and a set of universal duplex probes containing both FP and QP (Figure 1A). During the first and second cycle of amplification, one new synthetic chimeric DNA fragment with UT sequence at the 5' end can be generated from each initial template DNA molecule by PCR amplification (Figure 1B and 1C). During the annealing phase of the third cycle, FP hybridizes with these newly synthesized chimeric DNA strands, separating the QP from the FP (Figure 1D). Meanwhile, FP bound to the newly synthesized chimeric DNA strand can also function as a forward primer for synthesis of a new DNA strand based on the mother strand (Figure 1E). During the annealing period of the fourth cycle, the QP hybridizes with the chimeric mother strand having a FP at its 5' end, thus it can be hydrolyzed by the 5' exonuclease activity of *Taq *DNA polymerase (Figure 1Fa) or displaced (Figure 1Fb) by new strand synthesis. Either displacement or hydrolysis of the duplex probes will result in increasing fluorescent signal. Throughout the following PCR cycles, more and more DNA strands (Figure 1C and 1E) are employed for amplification and fluorescent signal emission. Unlike the signal generation mechanisms of TaqMan (hydrolysis) and double-stranded probes (strand displacement) at the first or second cycle, the AUDP PCR method can generate fluorescent signal in the third cycle through a combination of hydrolysis and strand displacement [26].

**Figure 2:**
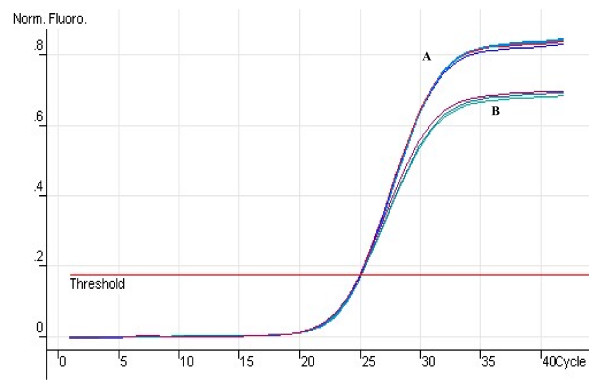
**Comparison of the amplification efficiency of AUDP PCR assays using *Taq *or *Pfu *polymerase**. (A) The amplification curves of *Taq *polymerase; (B) The amplification curves of *Pfu *polymerase.

To assess the ability of generating signal depending upon strand displacement in AUDP PCR assay, *Pfu *DNA polymerase without 5'→3' exonuclease activity was used to detect different target sequences, including *cp4EPSPS*, *Cry1A(b)*, *Lectin*, and the *Invertase I *gene. In the AUDP PCR assay for the *cp4EPSPS *gene, similar fluorescent curves were obtained with *Pfu *and *Taq *DNA polymerases, except that *Pfu *DNA polymerase resulted in reduction of fluorescent intensity (Figure 2). Similar results were also obtained in *Cry1A(b)*, *Lectin*, and *Invertase I *assays, indicating that DNA polymerases without 5'→3' exonuclease activity can still produce florescent signals by strand displacement of probes, verifying that the fluorescent signal emission of this assay can be independent of the 5'→3' exonuclease activity. The decreased fluorescence intensity is likely to be due to the different mechanisms of fluorescent signal generation between *Pfu *(strand displacement) and *Taq *(hydrolysis and strand displacement) in AUDP PCR.

### Reproducibility and repeatability of the AUDP PCR assays

After optimizing the concentration of primers, AUDP probes and MgCl_2_, the repeatability and reproducibility of CaMV35s promoter and NOS terminator AUDP PCR assays were tested using a series of dilutions of transgenic RRS genomic DNA solutions. The series were diluted to give a concentration range of 10–100,000 copies of soybean genomic DNA. Each serial dilution was assayed in triplicate per PCR run and runs were repeated three times. The repeatability standard deviation (SDr) and reproducibility standard deviation (SDR) of these two AUDP PCR assays were calculated. The SDr values of the CaMV35s and NOS PCR assays ranged from 0.09 to 0.17 and 0.08 to 0.19, respectively (Table 1); the SDR values ranged from 0.04 to 0.13 in the CaMV35s PCR assays, and from 0.05 to 0.14 in the NOS PCR assays (Table 1). This result indicates that the AUDP PCR assays were highly reproducible and could be used for further nucleic acids quantitative analysis.

### Simplex AUDP real-time PCR for GMO quantification

**Figure 3:**
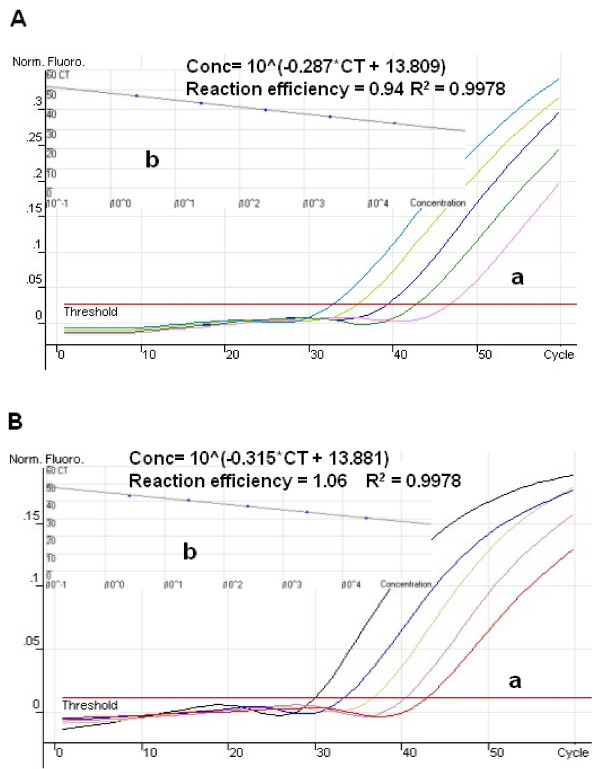
**The AUDP PCR assays for the CaMV35s promoter and NOS terminator of GM RRS**. (A) The standard curve of CaMV35s promoter quantification. a: Amplification plot generated by five dilutions (10-fold) of transgenic soybean ranging from 0.01 to 100 ng with CaMV35s promoter real-time PCR system; b: Initial DNA concentration versus Ct standard curve; (B) The standard curve of NOS terminator quantification. a: Amplification plot generated by five dilutions (10-fold) of transgenic soybean ranging from 0.01 to 100 ng with NOS terminator real-time PCR system; b: Initial DNA concentration versus Ct standard curve.

To prove the applicability of this method in DNA quantification, the AUDP PCR method was employed to quantify the CaMV35s promoter and NOS terminator contents in genetically modified RRS by relative quantitative method. Both standard curves of the endogenous and exogenous PCR system were constructed by serial dilution of the target DNA in separate tubes. Samples diluted from 100 ng to 10 pg of RRS DNA were employed by simplex CaMV35s and NOS AUDP PCR assay, respectively. The PCR efficiencies of CaMV35s and NOS assay were 0.94 and 1.06, respectively. The square regression coefficients (R^2^) of these two exogenous PCR assays were both 0.9978. Comparable results were also obtained from the AUDP PCR assay of soybean endogenous *Lectin *gene with a square regression coefficient of 0.9935 (Figure 3). The limits of detection (LODs) of CaMV35s and NOS AUDP assays were 10 copies. The excellent linearity between DNA concentrations and fluorescence values (Ct) visualized in the calibration curves and low LOD values indicate that the AUDP assays established in this study are well suited for quantitative measurements. Furthermore, more simplex AUDP PCR assays for GMOs quantification were developed, such as CaMV35s promoter and NOS terminator AUDP PCR assays for GMOs screening detection, RRS *Cp4-EPSPS *and Bt176 *Cry1A(b) *for gene-specific detection, and event-specific analyses for Bt11, MON863, MON810, and GA21 maize respectively. Totally twenty-four GM soybean and maize samples were quantified by the relative quantification method according to the ratio of transgene to total DNA quantity [28]. The bias of quantitative analysis was calculated based on the formula of ABS (Measurement Value - True value)/True value. For screening assays, three GM soybean samples with different GM contents (5.0%, 3.0%, and 1.0%) were quantified, and the results showed that all the GM soybean samples with different GM contents could be quantified with low bias value compared with their virtual GM contents ranging from 3.92% to 15.12%. As to gene-specific assays of *cp4EPSPS *and *Cry1A(b)*, the GM soybean and Bt176 maize samples with different GM contents (5.0%, 3.0%, and 1.0%) could be quantified with low bias (4.34%~19.22%) and standard deviation (0.04~0.20). For event-specific assays, the bias was between 3.53 and 21.13, and the SD between 0.05 and 0.40. All these indicate that the AUDP PCR assay can be successfully used in the simplex quantification of target DNAs. All the data is shown in Table 3.

### Duplex AUDP real-time PCR for GMO quantification

**Figure 4:**
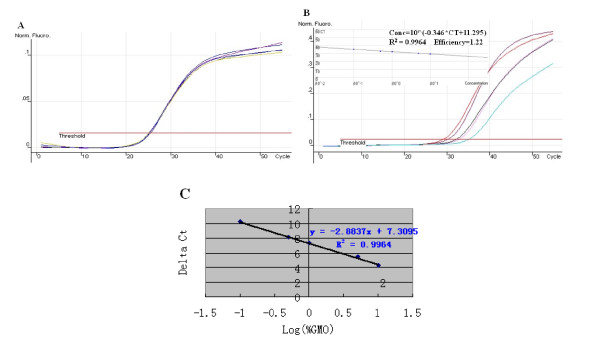
**Duplex fluorogenic PCR analysis for RRS detection employing the endogenous reference gene *Lectin *and exogenous gene *cp4EPSPS***. (A) Amplification plot of endogenous *Lectin *gene. Each dilution contained 100 ng of total soybean DNA. (B) Amplification plot of the transgenic *cp4EPSPS *gene, five different GM contents (10.0%, 5.0%, 1.0%, 0.5% and 0.1%) of soybean DNA that contained 10, 5, 1, 0.5 and 0.1 ng GM soybean DNA per reaction, respectively, were plotted as the initial RRS DNA concentration versus Ct standard curve (R^2 ^= 0.9964). (C) Standard curve, plotting log [GMOs concentration] versus change in threshold of detection (*Δ*Ct), R^2 ^= 0.9964.

Duplex PCR is less vulnerable to pipetting errors, less labor intensive, and less expensive than simplex PCR. In this study, duplex PCR systems for the quantitative analysis of RRS (*cp4EPSPS *and *Lectin *genes) and Bt176 maize (*Cry1A(b) *and *Invertase 1 *genes) were established, respectively. The sensitivity test for RRS indicated that the exogenous gene *cp4EPSPS *and endogenous *Lectin gene *could both be detected using as low as 10 pg, and which corresponded to 10 copies of haploid genomic DNA. The quantitative standard curve, which plots the log RRS concentration versus *Δ*Ct(Ct_cp4EPSPS_-Ct_Lectin_), was constructed using 100 ng of total DNA varying in GM contents from 0.1 to10% (w/w) according to the described method (*24*). The R^2 ^value of 0.9964 was obtained from the standard curve of the *cp4EPSPS *gene amplification. The standard curve generated by a plot of log GM content versus *Δ*Ct(Ct_cp4EPSPS_-Ct_Lectin_), indicated that the amplification efficiencies of the *cp4EPSPS *and *Lectin *genes were close and that the amount of the target transgene could be calculated based on the difference between the Ct values of the target and an endogenous reference gene (Figure 4).

As to the duplex PCR assay for Bt176 maize, the generated standard curve showed that the R^2 ^value was 0.9954. The tests of the three GM maize samples (5.0%, 3.0%, and 1.0%) showed that the mean GM content was of 4.69% to the 5.0% sample, 3.41% for the 3.0% sample, and 0.84% for the 1.0% sample. All above data indicated that the established duplex AUDP PCR assays are credible.

Gene expression analysis using AUDP PCR

**Figure 5:**
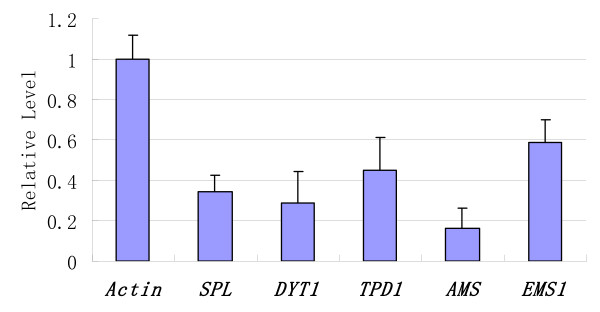
***Arabidopsis *gene expression analysis using AUDP PCR**. *SPL*: SPOROCYTELESS, *TPD1*: TAPETUM DETERMINANT1, *DYT1*: DYSFUNCTIONAL TAPETUM1, *AMS*: ABORTED MICROSPORES, *EMS1*: EXCESS MICROSPOROCYTES1.

To test the applicability of AUDP PCR in gene expression analysis, several *Arabidopsis *anther specific genes were selected, such as *SPL*, *DYT1*, *TPD1*, *AMS*, and *EMS1*, and their AUDP PCR probe and primers were designed. Gene expression in *Arabidopsis *flower bud was analyzed using AUDP real-time PCR according to the 2^-*ΔΔ*Ct ^methods [35]. In these optimized AUDP real-time PCR assays of these genes, the PCR amplified efficiencies were all above 0.90 and their amplified curves had good linearity. The results from AUDP real-time PCR assays indicated that these genes were all expressed in *Arabidopsis *flower bud, and the transcription level of these was shown in Figure 5, which is consistent with previous reports [36]. The results indicated that the AUDP PCR can be successfully applied to investigate different gene expression patterns using only one AUDP probe.

### Genotype discrimination using AUDP PCR

**Figure 6:**
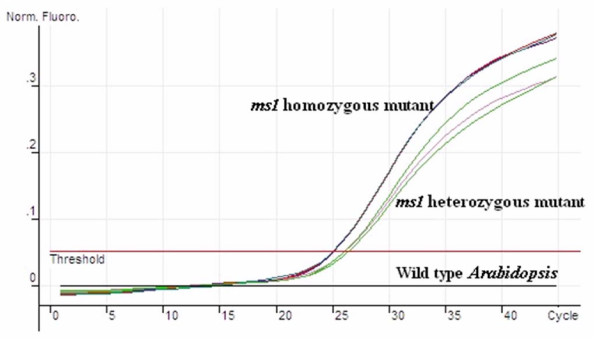
*Arabidopsis ms1* mutant genotype identification using AUDP PCR.

In the *ms1 *mutant of *Arabidopsis*, one "G" base was replaced by "A" in the genome, resulting in a loss of splicing of the first intron [34]. One AUDP PCR assay was employed to discriminate between the *ms1 *mutant and wild type. The AUDP PCR detection results showed that fluorescent signal could be obtained in the reaction of the homozygous and heterozygous *ms1 *plants. A mean Ct value of 25.29 was obtained in 100 ng homozygous *ms1 *genomic DNA reactions with 9 repeats, the mean Ct value of 26.41 in 100 ng heterozygous *ms1 *genomic DNA reactions (Figure 6), and the estimated copy number of homozygous *ms1 *mutated genomic DNA were about twice that of heterozygous *ms1 *mutated genomic DNA. From these results we believed that the AUDP PCR method can be further used for genotype discrimination.

### Analysis of longer target DNAs using AUDP PCR

From the unique principle of fluorescent signal generation in AUDP PCR, we hypothesize that this PCR assay has the ability to quantify target DNA with a range of template sizes. To test this, four AUDP PCR assays with different amplicon lengths (195, 546, 1032, and 1630 bp) were designed based on the pBI121 DNA. Likewise, four TaqMan and UT-PCR assays with similar amplicon sizes were designed, respectively, based on pBI121 sequence (Table 3). For each PCR assay chemistries, the PCR conditions were optimized with high efficiency (above 0.90), and the limits of detection (LOD) were tested. Fluorescent signals were obtained in all the four AUDP real-time PCR assays, regardless of amplified DNA length, and the LOD was 10 copies for the 195-bp and 546-bp amplicons, and 50 copies for the 1032-bp and 1630-bp amplicons (Table 4). However, fluorescent signals were detected for only 210-bp and 510-bp amplicons in TaqMan PCR assays, and no fluorescent signal was observed in the 992-bp and 1596-bp PCR assays. The LODs of the TaqMan PCR assays were 10 and 20 copies for the 210-bp and 510-bp amplicons, respectively (Table 4). Also we found that the UT-PCR assay could not be used for quantifying longer DNA fragments, as no fluorescent signal was observed in 1032-bp and 1630-bp amplicon assays (Table 4). These results demonstrate that the AUDP PCR assay described in this study can be successfully used for quantifying longer target DNAs with higher efficiency and sensitivity.

## Discussion

A new type of fluorescent real-time PCR has been developed in this study. The fluorescent probe in AUDP has a dual-role, it functions not only as the fluorescent probe, but also as an extension primer, which is different from other probes, such as TaqMan, UT probe, and aQRT PCR etc. This design enhances the intensity of the fluorescent signal intensity and allows for the quantitative analysis of longer DNA fragments by utilizing both the 5'→3' exonuclease activity and the 5'→3' DNA polymerase activity of the *Taq *DNA polymerase. In this quantitative PCR assay, the universal duplex probes hybridize to amplified product strands with attached UT sequences, thus permitting the use of a single set of duplex probes for multiple different target DNAs and greatly decreasing the cost of the assays. The present price of fluorescent probes is decuple more than that of AUDP PCR primers in general, which furthermore illustrates the lower cost of AUDP PCR compared with other fluorescent real-time assays except for the SybGreen I real-time PCR. Compared with the SybGreen I real-time PCR, the advantage of AUDP PCR is the applicability in duplex PCR analysis. However, the specificity of this PCR method depends upon target-specific primers. Thus the forward and reverse primers must be designed following the principles described above; otherwise, this PCR system may generate non-specific signals.

We established both simplex and duplex PCR methods for GMO quantification using the AUDP technique. The quantification capability of the simplex and duplex PCR assays has low sensitivity and wide dynamic range. In the duplex PCR system, different sets of duplex probes bearing different fluorescent reporters were designed for the exogenous and endogenous genes, allowing the simultaneous detection of multiple targets in one tube. The AUDP PCR technique could be also applied in gene expression analysis and genotype test. The results obtained in these experiments fully validate the extensive applicability and high accuracy of this technique for nucleic acid analysis. Furthermore, in all these tests, only one AUDP probe was used combined with more than 20 different primer pairs, which indicates that one AUDP probe can be used for different target DNA quantification reducing the cost of probes greatly and improved the PCR throughput.

Additionally, our results indicate that AUDP PCR assay can be used for longer fragments (at least as long as 1630 bp) with high efficiency (higher than 0.90) and low LOD (50 copies), this allows this method to be used to overcome difficulties in primer and probe design because of the limited length of the target DNA fragment in conventional assays.

## Conclusion

In this study, we present a novel real-time PCR technique, AUDP-PCR, which allows combined fluorescent signal generation. The applicability, reliability of AUDP PCR has been validated in GMOs quantification, gene expression, and genotype detection, and which will improve nucleic acids analysis with low costs and high throughput. We also expect that this technique will also be widely used in clinical diagnostics and pathogen identification for nucleic acids analysis in the near future.

## Methods

### Materials

Transgenic Bt176 and Bt11 maize developed and supplied by Syngenta Seeds, Inc. (Greensboro, NC, USA), GM maize lines (MON863, MON810, and GA21) and Roundup Ready soybean (RRS) developed and supplied by Monsanto Company (St Louis, MO), and non-transgenic maize and soybean (obtained from Shanghai local market) were used in this study. Wild type *Arabidopsis *Landsberg *erecta *and the *ms1 *mutant were from University of Nottingham. The pBI121 plasmid was purchased from BD Biosciences Clontech (Palo Alto, CA, USA).

### DNA extraction

Plant genomic DNA was extracted using Plant DNA Mini-Prep kit (Ruifeng Agro-tech Co., Ltd, Shanghai, China). Plasmid DNAs were isolated using Plasmid Mini Kit (Watson Biotechnologies, Inc, Shanghai, China). DNA quantity and quality was evaluated using absorbance measurements at 260 nm and 280 nm, and copy numbers were estimated by comparison to the size of plant and plasmid genomic DNA [27,28].

### RNA isolation and reverse transcription

*Arabidopsis *total RNA was isolated from flower buds using Trizol reagent (Invitrogen). After treatment with DNase (Promega), 0.3 μg RNA was used to synthesize the oligo(dT) primed first-strand cDNA using the ReverTra Ace-a-First Strand cDNA synthesis kit (TOYOBO). Three microliters of the reverse transcription products were subsequently used as template for PCR.

### Design of AUDP probes and primers

In this study, some candidate UT sequences were created to avoid significant sequence similarity between the UT sequence and any known genomic sequences especially those of pBI121, maize, canola and soybean, as well as the sequence of relevant transgenes and universal regulatory elements [18]. One UT sequence is selected and attached to the 5' end of the target specific primers. The universal duplex probes are composed of two complementary single-labeled oligonucleotides of differing length. The fluorescent probe (FP) labeled with a fluorophore at its 5' end has the UT nucleotide sequence with about 30 nucleotides (nt) in size, while the quenching probe (QP) is labeled with a non-fluorescence quencher (dabcy1) at its 3' end with about 20 nt that are complementary to the 5' end of FP.

The target-specific PCR primers were analyzed and designed by modifying the analysis results of Primer premier 5.0 (PREMIER Biosoft International, Inc.). The 5' end of one target-specific PCR primer was then attached to the UT sequence. In addition, the PCR primers and duplex probes were designed to minimize the formation of primer dimers, and other types of secondary structures between the primers and probes. Primer premier 5.0 (PREMIER Biosoft International, Inc) and Oligo 6 software (Molecular Biology Insights, Inc.) were used to analyze candidate primers to avoid these problems. The GC content for the primers, UT sequence, and duplex probes were 30–80%. The melting temperature (Tm) of FP/QP duplex probes was about 5~10°C higher than that of the target DNA specific primers. To optimize the GC content and Tm values of the target PCR primers, a linker region of about 3~6 nt was added between the UT sequence and the target-specific primer region. This linker also served as a flexible region for the primer during PCR.

### Primers and probes

Two sets of universal duplex probes were designed and used for more than 20 different target DNA/RNA sequences quantification in this study (Table 4). One was labeled with FAM fluorescent reporter and another with HEX fluorescent reporter.

**Table 1:** Primer pairs and universal duplex probes used in duplex PCR in this study^a^

Application	Target Gene	Orientation	Sequence (5'---3')	Amplicon Length (bp)
GMO quantification	CaMV35s promoter	Sense	taggaacaggcggcgacgagcgtgggacata*ATACAA*GACGCACAATCCCACTATC	68
		Anti-sense	CCTCTCCAAATGAAATGAACT	
	NOS terminator	Sense	taggaacaggcggcgacgagcgtgggacata*ATACAA*ATGTATAATTGCGGGACTCTA	52
		Anti-sense	TGACGTTATTTATGAGATGGGT	
	*cp4EPSPS*	Sense	taggaacaggcggcgacgagcgtgggacata *ATACAA*GGAACGTCTTCTTTTTCCACG	199
		Anti-sense	CTTATTGCATTTCATTCAAAATAAG	
	*Cry1A(b)*	Sense	taggaacaggcggcgacgagcgtgggacata*ATACAA*CCCCCCTCAGAACAACAA	56
		Anti-sense	GGCTCAGACGGTGGCT	
	*GA21*	Sense	CTTATCGTTATGCTATTTGCAACTTTAGA	112
		Anti-sense	atccttgtccgccgctgctcgcaccctgtat*ATA*TGGCTCGCGATCCTCCT	
	*Bt11*	Sense	taggaacaggcggcgacgagcgtgggacata*ATA*GCGGAACCCCTATTTGTTTATT	112
		Anti-sense	atccttgtccgccgctgctcgcaccctgtat*CGC*ACACCTACAGATTTTAGACCAA	
	*MON863*	Sense	taggaacaggcggcgacgagcgtgggacata *ATA*GGTTGGTTGGTGAGCCTAGTGA	130
		Anti-sense	CATCCGAACAAGTAGGGTCAAT	
	*MON810*	Sense	taggaacaggcggcgacgagcgtgggacata *ATA*TCTTTCAAGCCGAAGGTACATC	177
		Anti-sense	GCGAAGGTTATGAAGGACATAC	
Gene expression analysis	*Actin*	Sense	TGAAGTTCTGTTCCAGCCATCC	171
		Anti-sense	atccttgtccgccgctgctcgcaccctgtat*CGC*TTACTCATCCTATCACCAATCCC	
	*SPL*	Sense	TCCTCCGATGAACGGCTAC	70
		Anti-sense	atccttgtccgccgctgctcgcaccctgtatGGAAACCTTGGCTCCTCTG	
	*EMS1*	Sense	taggaacaggcggcgacgagcgtgggacata*TTT*CCTACATCACGGTTTCATCC	197
		Anti-sense	CTCGGGCACTCTGACCATAC	
	*AMS*	Sense	taggaacaggcggcgacgagcgtgggacata*AAA*TTGCGAATACAAACCAGGAG	102
		Anti-sense	ACCAGGCTGAGGTAGCGAGT	
	*TPD1*	Sense	CGTCTCCGTTGAAGCCTCC	188
		Anti-sense	atccttgtccgccgctgctcgcaccctgtat*AAA*CACCACTATGTCCGTACTCT	
	*DYT1*	Sense	CCAGTGAGGATGAGCCGTAG	133
		Anti-sense	atccttgtccgccgctgctcgcaccctgtat*AAT*GAGATCGCAGAGCCATAAGC	
Genotype identification	*ms1 *mutant	Sense	taggaacaggcggcgacgagcgtgggacata*TTA*CCGGTTTAAAGGCCAGa	251
		Anti-sense	ATGGCTCTCCTTTTGCTAC	
	*AUDP*	FP	FAM TAGGAACAGGCGGCGACGAGCGTGGGACATA	
		QP	AGTCGTCGCCGCCTGTTCCTA Dabcyl	

^a^UT sequences: taggaacaggcggcgacgagcgtgggacata, italicized nucleotide sequences are the linker region of the modified target specific primer between the UT sequence and target specific DNA primer.

**Table 2:** Primers and probes of real-time PCR assays using TaqMan, UT and AUDP based on the genomic DNA sequence of pBI121 (GenBank No. AF485783).

Fluorescent real-time PCR	Orientation	Sequence^a ^(5'---3')	Amplicon length (bp)
TaqMan	Sense	CCGCCTTTGAGTGAGCTGATA	210
	Anti-sense	TGTAGACAACATCCCCTCCCC	
	Probe	FAM CGCCGCAGCCGAACGACCGAG TAMRA	
	Sense	GTATTACCGCCTTTGAGTGAGCT	510
	Anti-sense	ATGACAATCAGCTACTTCACTGTTG	
	Probe	FAM CGCCGCAGCCGAACGACCGAG TAMRA	
	Sense	CCGCCTTTGAGTGAGCTGATA	992
	Anti-sense	CTCCGCTGGTCCGATTGAAC	
	Probe	FAM CGCCGCAGCCGAACGACCGAG TAMRA	
	Sense	CCGCCTTTGAGTGAGCTGATA	1596
	Anti-sense	AATGGTTTCTGACGTATGTGCTTAG	
	Probe	FAM CGCCGCAGCCGAACGACCGAG TAMRA	
UT^a^	Sense	taggaacaggcggcgacga*ATACAA *CATCCGCTTGCCCTCATC	195
	Anti-sense	TGCCAAAGGGTTCGTGTAG	
	Sense	taggaacaggcggcgacga *ATACAA *CATCCGCTTGCCCTCATC	546
	Anti-sense	GGGATAACGCAGGAAAGAACAT	
	Sense	CAGACGTGAAACCCAACATACC	1032
	Anti-sense	Taggaacaggcggcgacga *ATACAA *CCCAATAGCAGCCAGTCCCT	
	Sense	taggaacaggcggcgacga *ATACAA *TCTGCTGTAGTGAGTGGGTTGC	1630
	Anti-sense	GCCGATTGTCTGTTGTGCC	
	UT probe	FAM TCGTCGCCGCCTGTTCCTA TAMRA	
AUDP probe^b^	Sense	taggaacaggcggcgacgagcgtgggacata*AA*ACTCCATCCGCTTGCCCTCATC	195
	Anti-sense	TGCCAAAGGGTTCGTGTAG	
	Sense	taggaacaggcggcgacgagcgtgggacata*AAACTC*CATCCGCTTGCCCTCATC	546
	Anti-sense	GGGATAACGCAGGAAAGAACAT	
	Sense	CAGACGTGAAACCCAACATACC	1032
	Anti-sense	taggaacaggcggcgacgagcgtgggacata*AAACTC*CCCAATAGCAGCCAGTCCCT	
	Sense	taggaacaggcggcgacgagcgtgggacata*AAACCTC*TCTGCTGTAGTGAGTGGGTTGC	1630
	Anti-sense	GCCGATTGTCTGTTGTGCC	
	FP	FAM TAGGAACAGGCGGCGACGAGCGTGGGACATA	
	QP	AGTCGTCGCCGCCTGTTCCTA Dabcyl	

^a^UT sequence: taggaacaggcggcgacga, italicized nucleotide sequences are the linker region of the UT PCR primer between the UT sequence and target specific DNA primer.

^b^UT sequence: taggaacaggcggcgacgagcgtgggacata, italicized nucleotide sequences are the linker region of UT sequence and target specific DNA primer.

**Table 3:** Repeatability and Reproducibility of *CaMV35s *and *Nos *AUDP PCR assays

True copy number	Ct values			Mean	SD^r^	SD^R^
				
	1	2	3			
*CaMV35s*						

100000	30.43	30.24	30.09	30.25	0.17	0.08
10000	33.19	33.25	33.38	33.27	0.09	0.04
1000	36.59	36.75	36.48	36.61	0.14	0.10
100	40.05	40.16	39.97	40.06	0.10	0.13
10	43.11	42.99	43.16	43.07	0.09	0.09
*Nos*						

100000	30.22	30.12	30.28	30.21	0.08	0.05
10000	33.65	33.42	33.49	33.52	0.12	0.06
1000	36.94	36.58	36.84	36.79	0.19	0.11
100	40.66	40.98	40.75	40.80	0.17	0.09
10	43.19	43.56	43.31	43.35	0.19	0.14

SD^r^, repeatability standard deviation; SD^R^, reproducibility standard deviation.

**Table 4:** GM contents quantification of GM samples using AUDP PCR assays

**Target gene/event**	**Sample**	**Endogenous/exogenous**	**Ct**			**Mean copies**	**SD**	**GM content (%)**	**Bias**
							
			**Ct1**	**Ct2**	**Ct3**				
***Screen specific detection***									
**CaMV35s**	5%	exogenous	34.42	34.17	34.08	34.22	0.18	4.33	13.37
		endogenous	26.9	26.89	26.78	26.86	0.07		
	3%	exogenous	35.01	34.84	34.67	34.84	0.17	2.81	6.22
		endogenous	26.78	26.82	26.93	26.84	0.08		
	1%	exogenous	36.56	36.21	36.89	36.55	0.34	0.86	13.87
		endogenous	26.81	26.79	26.88	26.83	0.05		
**NOS**	5%	exogenous	32.46	32.38	32.1	32.31	0.19	4.80	3.92
		endogenous	26.91	26.73	26.83	26.82	0.09		
	3%	exogenous	33.21	33.32	33.07	33.2	0.13	2.67	10.92
		endogenous	26.81	26.92	26.97	26.9	0.08		
	1%	exogenous	34.37	34.29	34.78	34.48	0.26	1.15	15.12
		endogenous	26.93	26.99	27.13	27.02	0.10		
***Gene-specific detection***									
***Cp4EPSPS***	5%	exogenous	31.1	31.32	31.24	31.22	0.11	4.78	4.34
		endogenous	26.91	26.97	27.06	26.98	0.08		
	3%	exogenous	31.89	31.93	32.14	31.99	0.13	2.63	12.35
		endogenous	26.81	26.93	26.95	26.90	0.08		
	1%	exogenous	33.21	33.47	33.11	33.26	0.19	1.18	17.86
		endogenous	26.93	26.99	27.14	27.02	0.11		
***Cry1A(b)***	5%	exogenous	29.32	29.13	28.92	29.123	0.20	4.04	19.22
		endogenous	25.78	25.81	25.73	25.7	0.04		
	3%	exogenous	29.42	29.28	29.35	29.35	0.07	3.50	16.70
		endogenous	25.69	25.81	25.92	25.81	0.12		
	1%	exogenous	31.45	31.39	31.26	31.37	0.10	0.83	16.97
		endogenous	25.83	25.86	25.91	25.87	0.04		
***Event-specific detection***									
**MON863**	5%	exogenous	29.46	29.78	29.39	29.54	0.21	5.56	11.21
		endogenous	25.84	25.89	25.93	25.89	0.05		
	3%	exogenous	30.68	30.37	30.41	30.49	0.17	3.11	3.53
		endogenous	25.93	25.97	25.99	25.96	0.03		
	1%	exogenous	32.13	32.25	32.51	32.27	0.19	1.21	21.13
		endogenous	26.01	26.13	26.94	26.36	0.51		
**GA21**	5%	exogenous	29.81	29.78	29.53	29.71	0.15	5.62	12.37
		endogenous	26.07	26.09	25.95	26.04	0.08		
	3%	exogenous	30.39	30.48	30.57	30.48	0.09	2.81	6.29
		endogenous	25.92	25.79	25.84	25.85	0.07		
	1%	exogenous	31.49	31.41	32.13	31.68	0.40	1.12	11.55
		endogenous	25.79	25.63	25.89	25.77	0.13		
**MON810**	5%	exogenous	33.52	33.78	33.13	33.48	0.33	5.29	5.89
		endogenous	25.84	25.89	25.95	25.89	0.06		
	3%	exogenous	34.67	34.13	34.25	34.35	0.28	3.20	6.76
		endogenous	25.94	26.03	26.13	26.03	0.10		
	1%	exogenous	35.91	35.78	35.69	35.79	0.11	1.14	13.67
		endogenous	25.84	25.89	26.19	25.97	0.19		
**Bt11**	5%	exogenous	32.52	32.59	32.83	32.65	0.16	5.85	16.99
		endogenous	25.99	26.08	26.13	26.07	0.07		
	3%	exogenous	33.41	33.64	33.29	33.45	0.18	3.18	5.74
		endogenous	25.93	26.21	26.09	26.08	0.14		
	1%	exogenous	34.98	34.79	35.23	35.00	0.22	0.87	13.36
		endogenous	25.94	26.05	25.83	34.22	0.18		

**Table 5:** Comparison of the amplified length and sensitivity of TaqMan, UT and AUDP PCR

Amplicon Length	TaqMan PCR		UT-PCR		AUDP PCR	
			
	Amplification (Y/N)	Sensitivity (copies)	Amplification (Y/N)	Sensitivity (copies)	Amplification (Y/N)	Sensitivity (copies)
~200 bp	Y	10	Y	10	Y	10
~500 bp	Y	20	Y	20	Y	10
~1000 bp	N	--	N	--	Y	20
~1600 bp	N	--	N	--	Y	50

To reveal the advantages of AUDP technique, a series of target-specific primers were designed based on the plasmid DNA sequence of pBI121 to amplify target DNA sequences ranging from ~200 bp to ~1600 bp in length to validate the applicability of this real-time method. Meanwhile, TaqMan and UT-PCR primers and probes designed to amplify the DNA fragments with similar sizes to those of the AUDP PCR assays were produced for comparison. In the design of the four TaqMan real-time PCR assays with different sizes, the same TaqMan probe was designed for all the assays, the same sense primer was used except for the assay with 510 bp amplicon in size. The distances between the sense primer and TaqMan probe were less than 6 bp in these four real-time PCR assays according to the design principle of TaqMan PCR, and which might generate the similar efficiency of fluorescent signal production. The designed AUDP, UT, and TaqMan PCR primers and probes were listed in Table 3.

For GMOs quantification, the FAM-labeled probes were used for these exogenous genes, i.e. CaMV35s promoter, NOS terminator, *cp4EPSPS *gene, *Cry1A(b) *gene, and event-specific DNA fragments for Bt11, MON863, MON810 and GA21 maize, and the HEX-labeled probes were used for plant endogenous reference genes (*Lectin *and *Invertase I*), respectively. The primer pairs and probes are listed in Table 4.

To test the applicability of the AUDP PCR assay for gene expression and genotype discrimination, the FAM-labeled probe set were used for the following genes: *Actin*, *SPOROCYTELESS (SPL) *[29], *TAPETUM DETERMINANT1 (TPD1) *[30], *DYSFUNCTIONAL TAPETUM1 *(*DYT1) *[31], *ABORTED MICROSPORES *(*AMS) *[32], *EXCESS MICROSPOROCYTES1 (EMS1) *[33], and *ms1 *mutant [34]; their corresponding primers are also listed in Table 4.

### Real-time PCR assays

In this study, all the real-time PCR assays (AUDP, UT, and TaqMan) were performed with the similar program and PCR reagents except for the primers, probes, and Mg Cl_2 _conditions. The final volume of each real-time PCR assay was 25 μL, and the generated fluorescence was monitored in every PCR cycle at extension stage. The PCR assays contained the following mixture: 1 × PCR buffer, 100–300 nM primers, 200–400 nM FP, and 400–800 nM QP for different target sequences, 400 nM each of dATP, dGTP, dCTP, 800 nM dUTP, 1.5 U *Taq *DNA polymerase, 0.2 units of Amperase uracil N-glycosylase (UNG), and 6 mM MgCl_2_. In terms of UT-PCR and TaqMan PCR, the PCR conditions were similar to duplex probe PCR except for the concentrations of primers (100–300 nM), probes (600–900 nM), and MgCl_2_.

All real-time PCR assays were run using the following program: 2 min at 50°C, 10 min at 95°C, and 50 cycles of 30 s at 94°C, 30 s at 58°C, and 30 s at 72°C, except for the amplification of pBI121 with amplicon lengths greater than 500 bp, where 60 s at 72°C was used instead. Primers were synthesized by Invitrogen Co., Ltd (Shanghai, China), the fluorescent probes were synthesized by TaKaRa biotechnology Co., Ltd (Dalian, China), and other PCR reagents were purchased from Biocolors Co., Ltd (Shanghai, China).

All real-time PCR reactions were run on a fluorometric thermal cycler, Rotor-Gene 3000A (Corbett Research, Australia), and data was analyzed with the Rotor-Gene 3000A software (Corbett Research, Australia).

### AUDP PCR assay with Pfu DNA polymerase

To test the applicability of other DNA polymerase in AUDP real-time PCR assay, *Pfu *DNA polymerase lacking 5'→3' exonuclease activity was used in AUDP real-time PCR assay with the similar reaction condition to those mentioned above except that an equal amount of *Pfu *DNA polymerase and *Pfu *PCR buffer (BioColor Co., Ltd, Shanghai. China) were substituted for *Taq *DNA polymerase related reagents.

## Abbreviations

AUDP: Attached Universal Duplex Probe; UT: universal template; FP: fluorescent probe; QP: quenching probe; GMO: genetically modified organism; RRS: Roundup Ready soybean.

## Authors' contributions

LY designed and performed the experiments, and wrote the manuscript. WaL, conceived the experiments. LJ participated in the experiments of GMOs quantification using AUDP PCR. WeL conceived the design of AUDP probe. WC conceived the design of AUDP probe and the experiments. ZAW supplied the ms1 mutant, conceived genotyping and revised the manuscript. DZ conceived of this study, participated in its design and coordination and co-wrote the manuscript. All authors read and approved the final manuscript.
